# Morphological patterns of pneumonia in free-roaming cats: a necropsy and histopathological study

**DOI:** 10.3389/fvets.2026.1855974

**Published:** 2026-06-25

**Authors:** Rubia Avlade Guedes Sampaio, Gedean Galdino da Cruz Silva, Gabriela Santina Costa Henrique, Vinícius Mendes Gonçalves, Victória Maria Belarmino dos Santos, Wanessa Otoni de Souza Silva, Danielly Santos Silva, Markyson Tavares Linhares, Lucas Rannier Ribeiro Antonino Carvalho, Ricardo Barbosa Lucena

**Affiliations:** 1 Graduate Program in Animal Science and Health (PPGCSA), Universidade Federal de Campina Grande, Patos, Paraiba, Brazil; 2 Integrated Veterinary Center – CIVET, Campina Grande, Paraiba, Brazil; 3 Center for Agricultural Sciences, Universidade Federal da Paraíba, Areia, Brazil; 4 Department of Physiology and Pharmacology, Karolinska Institutet, Stockholm, Sweden; 5 PatoBiology Laboratory, Graduate Program in Animal Science, Universidade Federal da Paraíba, Areia, Brazil

**Keywords:** bronchopneumonia, coinfection, feline pneumonia, histologic pattern, infectious agents, necropsy, pathogens, pulmonary pathology

## Abstract

**Background:**

Pneumonia is a significant cause of morbidity and mortality in free-roaming domestic cats, particularly when associated with infectious agents and underlying systemic conditions. Post-mortem studies remain essential for characterizing pulmonary lesions and refining etiological interpretation, especially in under-monitored populations with limited or absent antemortem clinical information. In this context, recognition of morphologic patterns may help pathologists interpret pulmonary lesions more accurately and may provide clinicians with useful clinicopathological insights into concurrent disease, differential diagnoses, and likely causes of death.

**Objectives:**

To characterize the gross and histopathological patterns of pneumonia in free-roaming cats submitted for necropsy, to identify associated infectious agents when available, and to assess the clinicopathological relevance of pneumonia to outcome in a population in which clinical history is often incomplete.

**Methods:**

A retrospective necropsy-based study was conducted on feline pneumonia cases examined between 2014 and 2024. Gross and histopathological lung findings were reviewed and classified according to established morphological patterns of pneumonia. Available clinical records were also reviewed to assess antemortem clinical context, type of death, and the inferred relevance of pneumonia to outcome. Ancillary microbiological testing, including bacterial culture, was performed when lung samples were available.

**Results:**

Of 289 necropsied domestic cats, pneumonia was confirmed in 49 free-roaming cats. Most animals died spontaneously (47/49; 95.9%), whereas euthanasia was recorded in two cases (2/49; 4.1%). Antemortem clinical information was available for 31/49 cats (63.3%). The most frequent major morphologic category was bronchopneumonia (32/49; 65.3%), including 26 cases of conventional bronchopneumonia, three of aspiration pneumonia, and three of pleuropneumonia. Interstitial pneumonia/interstitial lung disease accounted for 8/49 cases (16.3%), including six cases of interstitial pneumonia and two of bronchointerstitial pneumonia. Pyogranulomatous pneumonia was identified in 5/49 cases (10.2%), granulomatous pneumonia in 3/49 (6.1%), and embolic pneumonia in 1/49 (2.0%). Pneumonia was interpreted as the primary cause of death or euthanasia in 30 cases (61.2%), a contributing factor in 15 (30.6%), and an incidental finding in 4 (8.2%). Infectious agents were heterogeneous, and bacterial growth was detected in a subset of cases, with *Staphylococcus* spp. isolated in 4/49 cats (8.2%), making it the most frequent bacterial genus identified.

**Conclusion:**

This study highlights the morphological diversity of feline pneumonia and reinforces the central role of post-mortem pathological examination in its classification and interpretation. Integration of gross, histopathological, clinical, and ancillary microbiological findings improves interpretation of the morphologic spectrum, etiologic associations, and clinicopathological relevance of feline pneumonia, particularly in free-roaming cats with incomplete clinical histories and frequent concurrent systemic disease.

## Background

The respiratory tract is highly vulnerable to harmful agents due to its essential role in gas exchange and continuous exposure to inhaled substances (1). In domestic felines, respiratory tract diseases represent a significant clinical concern because they are common, may cause substantial morbidity and mortality, and often present with nonspecific signs that overlap with other systemic or thoracic disorders (2). These conditions may result from the action of infectious agents, heart disease, neoplasms, trauma, toxins or irritating particles (3). Infectious diseases affecting the respiratory system may manifest as pneumonia when the pulmonary parenchyma is involved, although in some cases the condition remains confined to the upper airways (4).

In Brazil, free-roaming, semi-domiciled, and rescued cats are commonly encountered across different regions of the country (5). Because these animals are frequently exposed to overcrowding, poor sanitary conditions, environmental stressors, and unrestricted outdoor access, they may be at increased risk of respiratory infection and pathogen transmission (6, 7). In addition, infectious and immunosuppressive diseases are common in these populations and may predispose cats to pneumonia either directly or indirectly, particularly through impaired host defense or secondary bacterial infection (3).

Pneumonia encompasses a spectrum of inflammatory conditions affecting the pulmonary parenchyma, arising from both infectious and non-infectious processes. Infectious pneumonia may be caused by bacteria, viruses, fungi, or parasites, whereas non-infectious pneumonia is typically associated with aspiration or inhalation of toxic or irritating material (4, 8). Pneumonia may also be classified according to clinical and pathological features, including aspiration and the presence of associated foreign material (8). Commonly reported bacterial agents include *Staphylococcus* spp., *Escherichia coli*, and *Klebsiella* spp. (9).

Lower respiratory tract infections most commonly affect young cats, up to two years old (4). In older animals, they are less common and are usually associated with predisposing factors that lead to systemic immunosuppression, such as co-infections with the feline leukemia virus (FeLV) and feline immunodeficiency virus (FIV), endocrine and/or metabolic diseases, or immunosuppressive therapy with antineoplastic drugs or corticosteroids (10). Aspiration events can also lead to pneumonia, either as aseptic chemical pneumonitis caused by gastric acidity or as secondary bacterial pneumonia when infection develops (11).

Due to the overlap of clinical signs with other respiratory diseases, it is necessary to study and describe in detail the pathological characteristics of pneumonias in cats. Understanding the macroscopic and histopathological changes associated with the infectious agents involved not only allows differentiation from other lung diseases with similar presentations but also contributes to a more accurate diagnosis and appropriate therapeutic choice. Therefore, the aim of this study was to characterize the morphological patterns of pneumonia in domestic free-roaming cats based on necropsy and histopathological findings, and to describe associated etiological agents when identified through complementary diagnostic methods.

## Materials and methods

### Study design and case selection

A retrospective study was conducted based on necropsy records of domestic cats examined at the Veterinary Pathology Laboratory of the Universidade Federal da Paraíba (UFPB), over an 11-year period (2014–2024). All feline necropsy records from the study period were reviewed, and cases showing lesions involving the lower respiratory tract were identified. From this broader population, only cats classified as free-roaming were considered eligible for inclusion in the present study. Within this selected population, cases in which pneumonia was diagnosed based on gross and histopathological findings were included in the final analysis.

### Clinical data

Available antemortem clinical information was retrieved from submission records when available, including clinical history, antemortem clinical context, clinical signs, suspected diagnoses, relevant ancillary findings, and type of death (spontaneous death or euthanasia). Clinical data were limited in some cases because certain animals were found dead or submitted for necropsy without prior clinical evaluation.

### Study population

All cats included in this study were free-roaming, including semi-domiciled cats with unrestricted outdoor access, stray cats rescued by non-governmental organizations (NGOs), and owned cats with unrestricted outdoor access. Strictly domiciled/indoor cats were not included in the study. Cats were grouped by age as kittens (0–6 months), juveniles (>6 months–2 years), adults (>2–10 years), and geriatric cats (>10 years), adapted from Vogt et al. (12).

### Necropsy and tissue sampling

Complete necropsies were performed in all cases following standard procedures. Samples from the lungs and other major organs were routinely collected, fixed in 10% neutral buffered formalin, and processed for histopathological evaluation.

### Histopathology

Formalin-fixed tissues were routinely processed, embedded in paraffin wax, sectioned at 4 μm, and stained with hematoxylin and eosin (H&E). Lung lesions were evaluated and classified according to established morphological patterns of pneumonia (1, 13, 14). For analytical purposes, cases were grouped into major morphologic categories and subcategories based on their predominant gross and histopathological features, as summarized in Table 1.

**Table 1:** Morphologic criteria used to classify pneumonia patterns in free-roaming cats.

Major category	Subcategory	Main histopathological criteria*
Bronchopneumonia	Conventional bronchopneumonia	Airway-centered inflammation involving bronchi, bronchioles, and adjacent alveoli, with inflammatory exudate within airways and alveolar spaces.
	Aspiration pneumonia	Bronchopneumonia associated with aspirated intraluminal material within bronchi and bronchioles, often accompanied by necrosis and hemorrhage.
	Pleuropneumonia	Bronchopneumonia with marked fibrinous and neutrophilic extension to the pleura; isolated pleuritis was not included in this category.
Interstitial pneumonia/interstitial lung disease	Interstitial pneumonia	Predominant alveolar septal thickening with diffuse interstitial inflammatory involvement.
	Bronchointerstitial pneumonia	Concurrently prominent airway-centered and interstitial inflammatory lesions.
Pyogranulomatous pneumonia	–	Multifocal to coalescing, predominantly random and/or vasculocentric pyogranulomatous foci within the pulmonary parenchyma, composed of central neutrophilic aggregates surrounded by macrophages and plasma cells, frequently associated with vasculitis; pleural or serosal involvement may also be present.
Granulomatous pneumonia	–	Discrete granuloma formation with epithelioid macrophages and multinucleated giant cells.
Embolic pneumonia	–	Multifocal randomly distributed inflammatory foci associated with vascular lesions compatible with hematogenous dissemination.

*Classification was based on the predominant histopathological pattern, with selected subcategories retained when relevant to etiologic interpretation.

### Histochemical and ancillary diagnostic techniques

Additional histochemical stains were performed when indicated to aid in the identification of infectious agents. These included Ziehl–Neelsen for acid-fast bacteria, PAS and Grocott’s methenamine silver (GMS) for fungal organisms, and Alcian Blue for mucosubstances.

### Microbiological analyses

Bacteriological culture was performed on lung samples using standard microbiological techniques when fresh tissue was available. Bacterial isolates were identified based on colony morphology, Gram staining, and biochemical characteristics. In selected cases, identification was further supported by matrix-assisted laser desorption/ionization time-of-flight mass spectrometry (MALDI-TOF) (15).

### Retroviral testing

Testing for feline immunodeficiency virus (FIV) and feline leukemia virus (FeLV) was performed in a subset of cases using rapid serological (SNAP FIV/FeLV Combo, IDEXX Laboratories, Inc., USA) assays on antemortem blood samples collected during clinical evaluation, when available.

### Data collection and analysis

Data retrieved from necropsy records included age, sex, breed, type of death, antemortem clinical context, clinical history when available, gross and histopathological findings, and results of complementary diagnostic tests. Pneumonia cases were categorized according to morphological pattern, and descriptive statistical analysis was performed to determine absolute and relative frequencies. The inferred relevance of pneumonia to outcome was also descriptively analyzed.

### Clinical relevance of pneumonia to outcome

Based on the retrospective review of clinical records, necropsy findings, and histopathological evaluation, each pneumonia case was categorized according to its inferred relevance to clinical outcome as follows: primary cause of death/euthanasia, contributing factor, incidental finding, or undetermined. This classification was based on integrated review of the available antemortem clinical context, type of death, gross and histopathological severity and distribution of pulmonary lesions, and the presence of concurrent non-pulmonary diseases. Pneumonia was classified as the primary cause when pulmonary lesions were considered severe enough to account for death or euthanasia and no more significant competing fatal process was identified. It was considered a contributing factor when pneumonia was clinically and pathologically relevant but occurred in association with another major systemic or fatal condition. Cases were classified as incidental when pulmonary lesions were judged unlikely to have substantially influenced the outcome in the presence of a more significant non-pulmonary disease process. Cases were classified as undetermined when the available clinical and pathological information did not allow reliable attribution.

## Results

### Study population, clinical history, type of death, and inferred relevance of pneumonia to outcome

Over the 11-year study period, 289 necropsies were performed on domestic cats. Among these, 165 (57.1%) showed lesions involving the lower respiratory tract. According to the inclusion criteria adopted in this study, only free-roaming cats were evaluated in detail. Within this selected population, pneumonia was diagnosed in 49 cats, which constituted the study group. Most animals died spontaneously (47/49; 95.9%), whereas euthanasia was recorded in two cases (2/49; 4.1%). Antemortem clinical information was available for 31 cats (63.3%), while 18 cats (36.7%) were submitted directly for necropsy without clinically informative history or prior hospitalization records. Based on retrospective clinicopathological assessment, pneumonia was interpreted as the primary cause of death/euthanasia in 30 cases (61.2%), a contributing factor in 15 cases (30.6%), and an incidental finding in 4 cases (8.2%). Detailed case-level antemortem clinical context and inferred relevance of pneumonia to outcome are provided in Supplementary Table S1. These data indicate that pneumonia was clinically relevant in most cases and frequently represented either the primary cause of death/euthanasia or an important contributing lesion in free-roaming cats.

### Signalment and retroviral status

Most affected cats were mixed-breed animals (46/49; 93.9%), whereas Persian, Siamese, and Brazilian Shorthair cats accounted for one case each (2.0%). Adult cats were the most frequently affected age group (30/49; 61.2%), followed by kittens (8/49; 16.3%), juveniles (6/49; 12.2%), and geriatric cats (2/49; 4.1%). Age was not recorded in three cases (6.1%). Pneumonia occurred equally in males and females (24 cases each), with sex not reported in one case.

Of the 49 cats with pneumonia, 27 were tested for feline immunodeficiency virus (FIV) and/or feline leukemia virus (FeLV). Eight of these (29.6%) were positive for at least one retrovirus. FeLV infection alone was identified in six adult cats (22.2%), whereas concurrent FIV and FeLV infection was detected in two adult cats (7.4%). No cases of isolated FIV infection were recorded. The remaining cats were not tested because they were either submitted directly for post-mortem examination or were rescued in critical condition and died during initial clinical care, precluding further diagnostic evaluation.

### Concurrent conditions

Concurrent non-pulmonary conditions or potential predisposing factors identified in cats with pneumonia are summarized in Table 2. The most frequent were feline panleukopenia and septic conditions (3/49 each; 6.1%), followed by pyothorax, lymphoma, and systemic/cutaneous fungal disease (2/49 each; 4.1%). Less frequent but clinically relevant concurrent conditions (1/49; 2.0% each) included chronic kidney disease, hepatobiliary disease associated with *Platynosomum fastosum* infestation and cholangiocarcinoma, intestinal intussusception, fibrinous pericarditis, diaphragmatic hernia with pulmonary contusion, and nasal squamous cell carcinoma. Within the aspiration pneumonia subcategory, one case occurred in a kitten with feline panleukopenia and one in an adult cat after anesthesia or surgical intervention; in the remaining case, no specific predisposing condition could be established from the available records.

**Table 2:** Concurrent conditions or potential predisposing factors identified among 49 free-roaming cats with pneumonia.

System	Concurrent condition or potential predisposing factor	Cases (*n*)*	% of total (*n* = 49)
Multisystem (infectious)	Feline panleukopenia	3	6.1
	Septic conditions (including septicemia and septic shock)	3	6.1
Respiratory/pleural	Pyothorax	2	4.1
Hematopoietic/lymphoid	Lymphoma	2	4.1
Integumentary/multisystem	Systemic and/or cutaneous fungal disease	2	4.1
Urinary	Chronic kidney disease	1	2.0
Hepatobiliary	Hepatobiliary disease (*Platynosomum fastosum* infestation and cholangiocarcinoma)	1	2.0
Gastrointestinal	Intestinal intussusception	1	2.0
Cardiovascular	Fibrinous pericarditis	1	2.0
Traumatic thoracoabdominal	Diaphragmatic hernia with pulmonary contusion	1	2.0
Upper respiratory/integumentary	Nasal squamous cell carcinoma	1	2.0
Iatrogenic/perioperative	Anesthesia or surgical procedure (aspiration context)	1	2.0

*Categories were not mutually exclusive; some cats had more than one concurrent condition.

### Morphological patterns of pneumonia

Pneumonia cases were grouped into five major morphologic categories: bronchopneumonia, interstitial pneumonia/interstitial lung disease, pyogranulomatous pneumonia, granulomatous pneumonia, and embolic pneumonia. Bronchopneumonia was the predominant major category (32/49; 65.3%), comprising 26 cases of conventional bronchopneumonia, three cases of aspiration pneumonia, and three cases of bronchopneumonia with marked pleural involvement (pleuropneumonia). Interstitial pneumonia/interstitial lung disease accounted for 8/49 cases (16.3%), including six cases of interstitial pneumonia and two cases of bronchointerstitial pneumonia. Pyogranulomatous pneumonia was identified in five cases (10.2%), granulomatous pneumonia in three (6.1%), and embolic pneumonia in one (2.0%). The gross findings associated with each major category and selected subcategories are shown in Figure 1, and the corresponding histopathological findings are shown in Figure 2.

**Figure 1:**
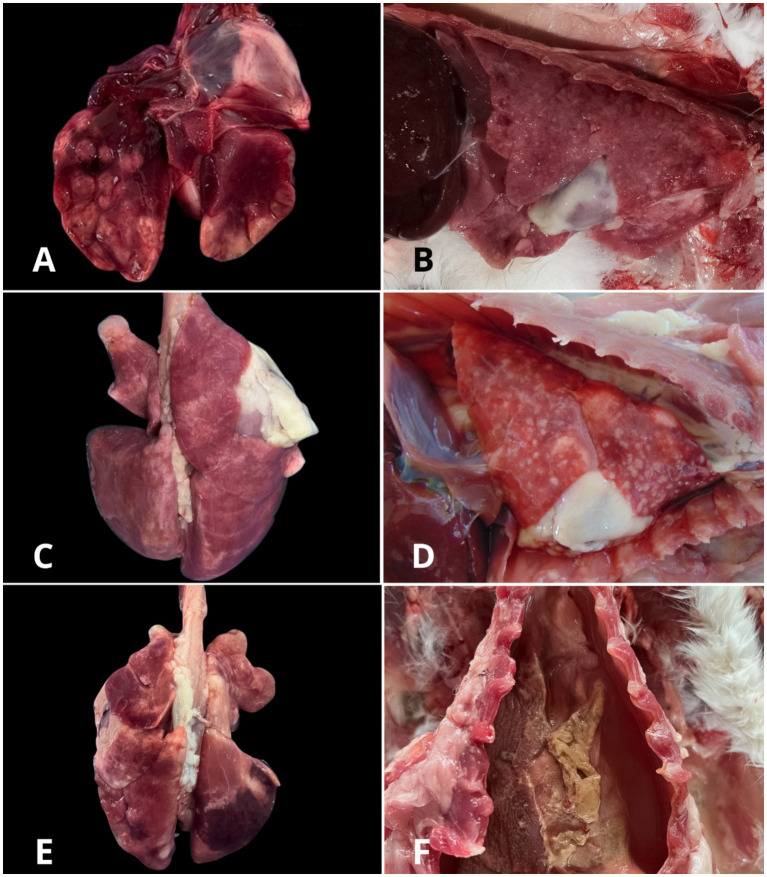
Gross pathological features of pneumonia in free-roaming cats. **(A)** Lungs markedly congested with multifocal to coalescing white-to-yellow nodules distributed throughout the pulmonary parenchyma, in a case of granulomatous pneumonia associated with *Nocardia* spp., in a 6-month-old female mixed-breed cat. **(B)** Lungs with multiple firm, white nodules diffusely distributed throughout the pulmonary parenchyma, in a case of granulomatous pneumonia in a 6-year-old female mixed-breed cat. Histopathological and histochemical findings supported mycobacterial infection. **(C)** Lungs mildly congested and uncollapsed, with evident rib impressions on the pleural surface, gross features associated with interstitial pneumonia, in a 5-year-old female mixed-breed cat. **(D)** Lung with multifocal pale and consolidated areas on a diffusely edematous background, corresponding histologically to bronchointerstitial pneumonia, in an adult female mixed-breed cat. **(E)** Lung with multifocal to coalescing white-to-hemorrhagic foci associated with parasitic infection, corresponding histologically to a mixed pattern of bronchopneumonia and interstitial pneumonia associated with *Aelurostrongylus abstrusus*, in a 6-year-old female mixed-breed cat. **(F)** Thoracic cavity containing abundant purulent exudate with associated pulmonary consolidation, consistent with fibrinosuppurative pleuropneumonia associated with *Staphylococcus aureus*, in a 5-year-old male mixed-breed cat.

**Figure 2:**
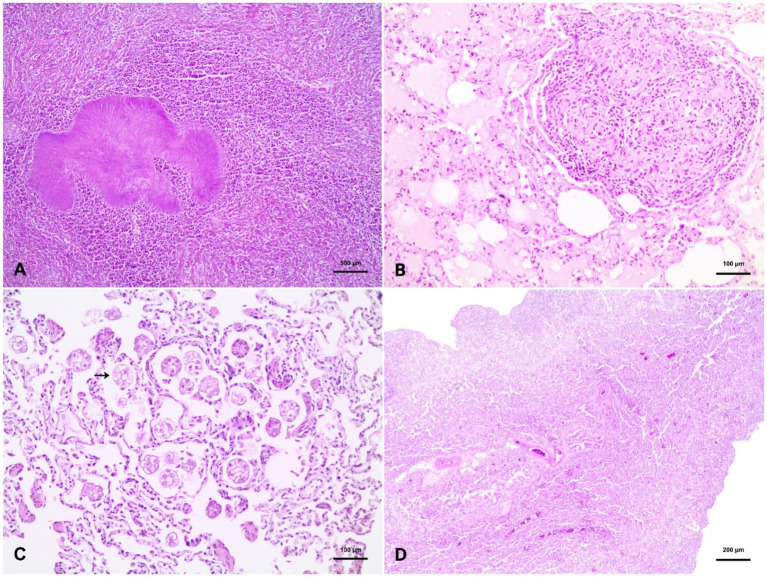
Histopathological features of pneumonia in free-roaming cats. **(A)** Granulomatous pneumonia associated with *Nocardia* spp. in a 6-month-old female mixed-breed cat. Focally extensive area of necrosis containing filamentous bacterial aggregates surrounded by *Splendore–Hoeppli* material. HE, 4×. **(B)** Granulomatous pneumonia associated with mycobacterial infection in a 6-year-old female mixed-breed cat. Well-formed granuloma composed of epithelioid macrophages and inflammatory cells. HE, 20×. **(C)** Parasitic pneumonia associated with *Aelurostrongylus abstrusus* in a 6-year-old female mixed-breed cat, characterized by embryonated parasite eggs (arrow) within alveolar spaces, accompanied by alveolar dilation, septal rupture, consistent with a mixed pattern of bronchopneumonia and interstitial pneumonia. HE, 20×. **(D)** Pleuropneumonia in a 5-year-old male mixed-breed cat. Extensive tissue necrosis extending from the lung parenchyma to the pleura, associated with abundant fibrin deposition and neutrophilic infiltrate. HE, 10 × .

Conventional bronchopneumonia (26/49) was characterized grossly by multifocal to coalescing firm white-yellow nodules, predominantly affecting the cranioventral lung lobes. Histologically, lesions consisted of mixed inflammatory infiltrates dominated by neutrophils and macrophages, commonly associated with fibrin deposition. Extensive parenchymal necrosis was observed in three cases, whereas interstitial septal thickening and bronchial epithelial loss were identified in isolated cases. Type II pneumocyte hyperplasia was observed in four cases. In a subset of cases, a fibrinosuppurative necrohemorrhagic pattern was present.

Aspiration pneumonia, treated as a subcategory of bronchopneumonia, was identified in three cats. These cases were distinguished from conventional bronchopneumonia by the presence of abundant amorphous aspirated intraluminal material within bronchi and bronchioles, associated with multifocal to coalescing hemorrhage and parenchymal necrosis. Grossly, lesions consisted of multifocal red and white foci, predominantly in the cranial lung lobes. In accordance with the final etiological classification, bacterial involvement in aspiration pneumonia was interpreted as secondary in some cases rather than primary.

Pleuropneumonia, treated as a subcategory of bronchopneumonia with marked pleural extension, was identified in three mixed-breed cats. Grossly, there was abundant fibrinosuppurative exudate within the thoracic cavity, extending to the pericardial sac, with multifocal fibrinous plaques on the parietal pleura and pulmonary areas of necrosis bridging the parenchyma and pleural surface. The pulmonary lesions were predominantly distributed in an airway-centered and cranioventral pattern, consistent with bronchopneumonia, with subsequent extension to the pleura rather than primary pleural disease. Histologically, lesions consisted of suppurative to fibrinosuppurative inflammation centered on bronchi, bronchioles, and adjacent alveoli, with extension into the pleura, associated with extensive fibrin deposition and dense neutrophilic infiltrates affecting both pleura and lung parenchyma, consistent with fibrinosuppurative pleuropneumonia. This distribution supported classification within the bronchopneumonia spectrum and helped distinguish these cases from processes primarily centered in the pleural cavity, such as pyothorax or pleural extension secondary to penetrating foreign material. *Staphylococcus aureus* was isolated in one case, whereas the remaining cases were culture-negative.

Within the interstitial pneumonia/interstitial lung disease group, interstitial pneumonia (6/49) commonly showed, on gross examination, diffuse pulmonary edema, mild congestion, evident rib impressions on the pleural surface, and failure of lung collapse. Histologically, these cases showed diffuse thickening of alveolar septa associated with pneumocyte necrosis, fibrin deposition, and inflammatory infiltrates predominantly affecting the interstitium and composed mainly of macrophages and neutrophils. In some cases, inflammatory cells were also present within alveolar spaces. One case exhibited a mixed bronchopneumonic and interstitial pattern and was associated with parasitic infection by nematodes morphologically compatible with *Aelurostrongylus abstrusus*, characterized by numerous embryonated eggs within alveolar spaces, alveolar dilatation, septal rupture, and interstitial inflammation. This same case also yielded *Klebsiella aerogenes*, supporting its interpretation as a mixed parasitic-bacterial pneumonia rather than a purely interstitial lesion.

Bronchointerstitial pneumonia, included within the broader interstitial pneumonia/interstitial lung disease category, was identified in two cases. Grossly, the lungs were diffusely edematous, with mild multifocal areas of increased firmness, particularly in peribronchiolar regions. Histologically, lesions involved both airway-centered and interstitial compartments and consisted of mixed infiltrates of lymphocytes, macrophages, plasma cells, and neutrophils surrounding bronchi and bronchioles and infiltrating alveolar septa. Intra-alveolar basophilic granular material was observed in some areas, but its nature could not be determined with certainty on hematoxylin and eosin sections alone. Hemosiderin-laden macrophages were also present in some areas. Classification was based on the concurrent prominence of airway-centered and interstitial lesions rather than on the interpretation of this granular material.

Pyogranulomatous pneumonia was identified in five cats and was considered morphologically compatible with feline infectious peritonitis (FIP). Grossly, these cases showed multifocal to coalescing, predominantly random nodular lesions throughout the pulmonary parenchyma, in some cases with associated pleural involvement. Histologically, they were characterized by multifocal to coalescing pyogranulomatous foci composed of central neutrophilic aggregates surrounded by macrophages and plasma cells, frequently accompanied by congestion, edema, emphysema, and neutrophil-rich vasculitis. No fungal or parasitic organisms were identified in these lesions on H&E or special stains.

Granulomatous pneumonia was identified in three cats and comprised three distinct etiologies. One case was associated with *Cryptococcus* spp. and was characterized grossly by multifocal to coalescing whitish pulmonary nodules. Histologically, well-formed granulomas composed predominantly of epithelioid macrophages and multinucleated giant cells contained numerous round to oval yeast-like organisms measuring approximately 5–15 μm, with a thin basophilic cell wall, a non-staining pericellular halo, and occasional narrow-based budding. These organisms stained positively with PAS and GMS, and fungal culture confirmed the diagnosis. The second case was associated with mycobacterial infection and was characterized by multiple randomly distributed granulomas composed predominantly of epithelioid macrophages and multinucleated giant cells. Ziehl–Neelsen staining demonstrated numerous intralesional acid-fast bacilli, confirming mycobacterial infection. The third case was associated with *Nocardia* spp. and was characterized by multiple coalescing granulomas with central necrosis containing filamentous organisms surrounded by radiating eosinophilic material consistent with the Splendore–Hoeppli phenomenon; microbiological culture confirmed *Nocardia* spp.

Embolic pneumonia was identified in a single one-year-old mixed-breed male cat with small intestinal intussusception. Grossly, the lungs showed multifocal, poorly demarcated reddish to pale tan foci scattered throughout the parenchyma. Histologically, there were multiple necrosuppurative foci of variable size, interpreted in the context of a septic embolic pattern.

### Etiology and microbiological findings

Fresh lung tissue for bacteriological culture was available in 28/49 cases, and culture was performed in all of these cases. Bacterial growth was detected in a subset of cultured cases, and the isolates were heterogeneous (Table 3), showing variable associations with the major morphologic categories and selected subcategories of pneumonia. The most frequently isolated bacteria were *Staphylococcus* spp. (4 cases) and *Staphylococcus aureus* (3 cases), both identified exclusively in adult and geriatric cats. In contrast, *Escherichia coli* (3 cases) was identified exclusively in kittens. Other bacterial isolates included *Klebsiella* spp. (2 cases), *Klebsiella pneumoniae* (1 case), *Klebsiella aerogenes* (1 case), *Enterobacter* spp. (1 case), *Pseudomonas* spp. (1 case), and *Nocardia* spp. (1 case).

**Table 3:** Frequency and age distribution of bacterial isolates in free-roaming cats with pneumonia.

Bacterial agent	Number of cases	Age group
*Staphylococcus* spp.	4	Adults/geriatrics
*Staphylococcus aureus*	3	Adults/geriatrics
*Escherichia coli*	3	Kittens
*Klebsiella* spp.	2	Adults/juveniles
*Klebsiella pneumoniae*	1	Juveniles
*Klebsiella aerogenes*	1	Juveniles
*Enterobacter* spp.	1	Juveniles
*Pseudomonas* spp.	1	Juveniles
*Nocardia* spp.	1	Juveniles

A mixed bacterial infection was observed in one case, with concurrent isolation of *Klebsiella pneumoniae*, *Pseudomonas* spp., and *Staphylococcus aureus*. Among the less frequent isolates, *Nocardia* spp. was identified in a case of granulomatous pneumonia characterized histologically by multiple coalescing granulomas with central necrosis and *Splendore–Hoeppli* material. *Staphylococcus aureus* was also recovered from one case of pleuropneumonia. The remaining culture-positive cases yielded heterogeneous bacterial isolates without a predominant additional pattern.

A single case of granulomatous pneumonia was associated with fungal infection, with histopathological features consistent with *Cryptococcus* spp. The fungal organisms stained positively with PAS and GMS, and fungal isolation was also obtained. In addition, one case of granulomatous pneumonia showed histopathological features consistent with mycobacterial infection, supported by Ziehl–Neelsen staining.

Parasitic infection was identified in one case showing a mixed pattern of interstitial pneumonia and bronchopneumonia, in which numerous nematode eggs morphologically compatible with *Aelurostrongylus abstrusus* were observed within alveolar spaces. This case was also associated with secondary bacterial infection, with isolation of *Klebsiella aerogenes*.

## Discussion

This study of 49 free-roaming cats demonstrates that the diversity and overlap of post-mortem morphological patterns of pneumonia reflect complex interactions among infectious agents, host factors, and concurrent systemic disease. In this population, such clinicopathological heterogeneity is especially relevant because many animals were presented with limited antemortem information, advanced multisystem disease, or late-stage respiratory compromise. Moreover, the finding that pneumonia represented the primary cause of death or euthanasia in most cases, but was contributory or incidental in others, emphasizes that pulmonary lesions in free-roaming cats should not be interpreted in isolation. Instead, these results support the clinicopathological value of necropsy-based pattern recognition for distinguishing primary pulmonary disease from secondary or incidental pneumonia in cats with incomplete histories and multiple comorbidities.

Most cats affected by pneumonia were mixed-breed animals (93.9%). This proportion mirrors the overall population of cats submitted for necropsy at the service, regardless of cause of death, indicating that this finding likely reflects the underlying feline population structure rather than a breed-related predisposition (2). Moreover, breed predisposition has not been demonstrated for diseases of the lower respiratory tract in cats (16).

Breed-associated susceptibility to respiratory disease has been documented only in specific contexts, such as *Mycobacterium avium* infection in Abyssinian cats as part of systemic disease (17) and a higher frequency of upper respiratory tract aspergillosis in brachycephalic purebred cats (18). However, none of these breeds were represented in the present study.

Regarding sex distribution, bronchopneumonia affected male and female cats similarly in the present study, indicating that sex was not a relevant determinant for the occurrence of pneumonia in this cohort. This finding contrasts with previous reports that identified male cats as being up to 2.4 times more likely to develop pneumonia than females (19).

The absence of a sex-related predisposition in the present study is likely influenced by the epidemiological profile of the population evaluated, which included a high proportion of stray and shelter cats, as well as animals originating from environments characterized by high population density and limited sanitary control. Such conditions are associated with increased exposure to infectious agents, stress-related immunosuppression, and frequent co-infections, factors that may override sex-related behavioral differences traditionally associated with pneumonia risk (10, 20–22).

The predominance of adult cats in this study likely reflects cumulative exposure to infectious agents and a higher frequency of underlying or concurrent conditions, including retroviral infections, rather than age-specific behavioral factors alone (21, 23). In the present study, a proportion of cats tested positive for feline immunodeficiency virus (FIV) and/or feline leukemia virus (FeLV), agents known to impair host immune responses and compromise pathogen clearance (9, 10). Such immunosuppression may facilitate the development of pneumonia either directly or through secondary bacterial colonization following primary viral infection. Although viral pneumonia and viral–bacterial co-infections are well recognized in cats, data addressing concurrent infections in feline pneumonia remain limited (9). In addition, FeLV-associated immunosuppression has been linked to the development of neoplastic diseases, including lymphoma, which may further predispose affected cats to severe pulmonary infections (24).

Bronchopneumonia, including the aspiration and pleuropneumonia subcategories, was the predominant major morphologic category identified in the present study. Conventional bronchopneumonia accounted for most cases within this group, which is consistent with previous retrospective necropsy-based investigations in cats and supports the view that bronchocentric inflammatory disease remains the dominant pulmonary pattern in free-roaming populations (16). Although bronchopneumonia often shows a cranioventral distribution, lesions may also extend to the cranial portions of other lung lobes, including the diaphragmatic lobe (1), a feature that was also observed in a subset of cases in the present study. From a clinical perspective, the predominance of bronchopneumonia indicates that bacterial and aspiration-related processes should remain high on the list of differentials in free-roaming cats with respiratory compromise, particularly when concurrent systemic disease is present.

Several concurrent conditions identified in cats with bronchopneumonia, including feline panleukopenia, trauma with or without septicemia, pyothorax, lymphoma, and chronic kidney disease, likely acted as predisposing or aggravating factors rather than primary causes. Chronic kidney disease, for example, is known to influence pulmonary homeostasis through disturbances in fluid balance, acid–base regulation, and vascular dynamics, potentially contributing to pulmonary congestion, edema, and increased susceptibility to infection (25). In addition, conditions such as retroviral infection and lymphoma may impair immune function, facilitating the development or progression of bacterial pneumonia (9, 24). Taken together, these findings suggest that recognition of bronchopneumonia at necropsy should prompt careful investigation for underlying comorbidities, particularly retroviral infection, lymphoid neoplasia, septic conditions, and other systemic disorders that may modify both lesion severity and clinical outcome.

The five cases of pyogranulomatous pneumonia in this study (10.2% of total cases) were interpreted as morphologically compatible with FIP based on the presence of predominantly random and vasculocentric pyogranulomatous inflammation centered on and around blood vessels, frequently accompanied by vasculitis. In some cases, similar lesions were also observed in other serosal surfaces and/or organs, further supporting this interpretation. In the absence of demonstrable fungal or parasitic organisms on H&E and special stains, FIP was considered the principal differential diagnosis for this pattern. This vasculocentric pattern is clinically relevant because it helps distinguish FIP-compatible pulmonary lesions from bacterial serositis, which typically shows a more predominantly suppurative inflammatory response (1). Although the cases in the present study lacked ancillary confirmation, their thoracic presentation appeared more consistent with a non-effusive or mixed form of FIP than with classic effusive disease. This contrasts with the broader respiratory spectrum described by Slaviero et al. (2024), in which pleural lesions and thoracic effusions were prominent, while also reinforcing that FIP may involve not only pyogranulomatous pneumonia but also pleural and pleuropneumonic lesions in the respiratory tract. However, in the absence of ancillary confirmation, these cases were considered compatible with, rather than definitively diagnostic of, FIP. For clinicians and pathologists, recognition of this pattern should prompt targeted investigation for FIP, including careful assessment of extra-pulmonary lesions and, whenever feasible, confirmatory ancillary tests such as immunohistochemistry or molecular assays. In contrast, bronchopneumonia encompassed a wider range of etiologies, particularly bacterial infections. Additionally, fibrinosuppurative inflammatory patterns were frequently observed histologically in this study, a finding that has been described in feline pneumonias and may reflect acute or peracute disease courses (14), even when an overt secondary infection is not documented.

As a subcategory within the broader bronchopneumonia group, aspiration pneumonia occurred sporadically and was not consistently associated with identifiable predisposing conditions. Although a viral enteric disease was present in one animal, in most cases no clear underlying factor could be established based on the available records. This finding aligns with previous reports indicating that aspiration pneumonia in cats may arise from a variety of clinical contexts, including gastrointestinal disease, neurological dysfunction, recumbency, or peri-anesthetic events, and may remain etiologically obscure in necropsy-based investigations (26). Clinically, this reinforces the need to consider aspiration whenever acute respiratory compromise develops in cats with recent non-pulmonary illness or procedural intervention, even when the precipitating event is not clearly documented.

Similarly, pleuropneumonia was interpreted as bronchopneumonia with marked pleural extension rather than as an isolated pleural process, a distinction that better reflects the concurrent involvement of pulmonary parenchyma and pleura in these cases. This distinction is also clinically important because pleural extension generally reflects more severe disease and may be associated with pleural exudation or pyothorax, which in comparable antemortem cases may justify pleural fluid sampling and, when indicated, thoracic drainage (1, 27).

Interstitial pneumonia/interstitial lung disease represented the second most frequent major morphologic category in this study and included both diffuse interstitial lesions and cases with concurrently prominent airway-centered and interstitial inflammation. In this context, bronchointerstitial pneumonia was interpreted within the broader interstitial lung disease spectrum rather than as an entirely separate category.

Like aspiration pneumonia, fungal and parasitic pneumonias were less frequently identified than bacterial and viral infections, consistent with previous necropsy-based studies (16, 19). Infection with *Aelurostrongylus abstrusus* may range from subclinical to severe pulmonary disease, particularly when associated with concurrent infections or immunosuppression. In the present study, the parasitic pneumonia case occurred in a FeLV-positive cat and was associated with secondary bacterial infection (*Klebsiella aerogenes*), supporting a multifactorial pathogenesis. FeLV compromises the immune system, making the animal more susceptible to infections and complications (23). Histopathologically, this case was classified as a mixed pattern of interstitial pneumonia and bronchopneumonia, reflecting the coexistence of airway-centered and interstitial lesions associated with parasitic structures. Although previous reports have categorized parasitic pneumonia as granulomatous (14), the lesion in the present case was not predominantly characterized by discrete nodular granulomas. Rather, the dominant microscopic pattern consisted of concurrent airway-centered and interstitial inflammation associated with parasitic structures, and the case was therefore classified according to its predominant morphologic features, without excluding the possibility of a concurrent macrophage-rich inflammatory component. In addition, the isolation of *Klebsiella aerogenes* in this case suggests that concurrent bacterial infection may have contributed to the mixed inflammatory pattern and modified lesion expression. This finding also highlights that the identification of parasitic structures in the lung should not preclude investigation for concomitant bacterial infection in cats with pneumonia. This approach emphasizes that lesion classification should be based primarily on the dominant histopathological pattern rather than on gross appearance alone, particularly given the variable inflammatory responses associated with parasitic infections. Fungal pneumonia was infrequently identified in this study, with a single case associated with *Cryptococcus* spp. Colonization of the respiratory tract by this organism may occur without overt clinical signs, and in the present case, no respiratory symptoms were reported, similar to that reported in other studies of the respiratory form of this infection (28, 29). The animal was presented for clinical evaluation only after the onset of neurological involvement. The thick polysaccharide capsule characteristic of this organism interferes with phagocytic activity and modulates host immune responses, which may contribute to a relatively mild or localized inflammatory reaction in affected pulmonary tissues (1).

Published data on feline pneumonia in Brazil remain limited, particularly in necropsy-based studies. In southern Brazil, bacterial and viral infections predominated, whereas fungal and parasitic pneumonias were less frequent (14), suggesting that the identification of only one parasitic and one fungal pulmonary case in the present cohort is not necessarily unexpected, even in a free-roaming population. In Brazil, *Aelurostrongylus abstrusus* pneumonia has been documented in retrospective studies and case series (30, 31), indicating that verminous pneumonia is a recognized but relatively uncommon cause of feline pulmonary disease rather than an exceptional finding. Likewise, fungal respiratory disease in cats appears to be sporadic in the literature, with reports including cryptococcosis (29) and invasive aspergillosis (32), while mycobacterial infection has also been documented in domestic cats from northeastern Brazil (33). Taken together, these observations suggest that the pathogens identified in the present study are not necessarily novel in the regional setting, but that their frequency in Brazilian feline populations remains poorly characterized, especially in free-roaming cats.

A single case of embolic pneumonia was identified in a one-year-old male mixed-breed cat, characterized by multiple randomly distributed pulmonary lesions of variable size, interpreted in the context of hematogenous dissemination of inflammatory material. Embolic pneumonia is characterized by multifocal pulmonary inflammatory lesions of variable size, often associated with necrosis and abscess formation, and vascular involvement, reflecting hematogenous spread (1). In the present case, intestinal intussusception was identified as a concurrent condition. This gastrointestinal disorder may lead to compromised intestinal perfusion, mucosal injury, and, in advanced stages, ischemia and necrosis, which can facilitate bacterial translocation and endotoxemia (34). In this cat, the affected intestinal segment showed marked necrosis associated with intralesional bacterial aggregates, indicating severe intestinal injury and suggesting a possible source of systemic bacterial dissemination. Although a direct causal relationship between the intestinal lesion and the pulmonary embolic pattern cannot be established, such systemic events are recognized as potential sources of septic emboli that may lodge in the pulmonary vasculature and give rise to lesions consistent with embolic pneumonia (1).

Bacterial culture was performed on lung samples collected during post-mortem examination when material was available. Bacterial growth was detected in a subset of cases, while no growth was obtained in others. Bacterial isolates were heterogeneous; however, *Staphylococcus* spp. and *S. aureus* were the most frequently identified agents, particularly in adult and geriatric cats, whereas *E. coli* was restricted to kittens. In several cases, pneumonia was associated with non-bacterial conditions identified histologically, including viral, parasitic, neoplastic, or systemic diseases. The absence of bacterial growth in some cases may be related to prior antimicrobial therapy or to pneumonia not primarily associated with bacterial infection.

Although *Staphylococcus* spp. and *Staphylococcus aureus* were among the most frequently isolated bacteria in the present study, their etiologic significance should be interpreted cautiously. In the present study, these isolates were obtained from post-mortem lung tissue and were interpreted in conjunction with the gross and histopathological pulmonary lesions, as well as the overall clinicopathological context. Thus, while staphylococci may have acted as opportunistic or secondary pathogens in some cases, their recovery alone was not considered sufficient to establish primary causation (3, 9). In addition, *Staphylococcus* spp. have been reported as predominant aerobic isolates in feline pleural infections, supporting their relevance in lower respiratory tract disease in cats (27). The prognosis of pneumonia in cats is influenced by multiple factors, including disease severity, host immunocompetence, and pathogen virulence (26). In the present study, bacterial growth, when detected, most often involved a single bacterial species; however, mixed bacterial infection was identified in one case, with concurrent isolation of *Klebsiella* spp., *Pseudomonas* spp., and *S. aureus*.

Disruption of respiratory defenses in primary infections may facilitate secondary bacterial colonization. Although *E. coli* and *K. pneumoniae* are among the most frequently reported bacterial agents in feline pneumonia, a broad range of opportunistic bacteria may be recovered from the lower respiratory tract of affected cats (20). In this context, the isolation of *E. coli*, *Enterobacter* spp., and *Staphylococcus* spp. in the present study is consistent with previous reports. Additionally, *Staphylococcus* spp. have been described as predominant aerobic isolates in feline pleural infections, reinforcing their relevance in lower respiratory tract disease in cats (27).

Granulomatous pneumonia in the present study comprised distinct etiologies, including fungal, mycobacterial, and nocardial infection. The cryptococcal case was characterized by well-formed granulomas containing numerous yeast-like organisms, whereas the mycobacterial case showed multiple granulomas with Ziehl–Neelsen-positive acid-fast bacilli. In addition, one case associated with *Nocardia* spp. was characterized as granulomatous pneumonia because the predominant lesion consisted of multiple coalescing granulomas with central necrosis and Splendore–Hoeppli material, rather than a predominantly airway-centered suppurative pattern. Although nocardiosis is uncommon in cats, it has been reported in association with pyothorax, pleuritis, and pulmonary infection (1). These findings highlight the importance of careful histopathological assessment and ancillary testing in cases of granulomatous inflammation, particularly when distinguishing among fungal, mycobacterial, and bacterial causes (30).

Taken together, these findings indicate that pulmonary lesions in free-roaming cats frequently reflect multisystem disease rather than isolated respiratory pathology. For clinicians and referring veterinarians, this means that the respiratory system should not be overlooked even in cats presented primarily for apparently non-respiratory conditions, such as diarrhea, neoplasia, cutaneous disease, or other systemic disorders, particularly when history is incomplete or follow-up is limited. In such cases, pneumonia may be clinically silent or masked by extrapulmonary disease, yet still represent an important secondary or incidental lesion that contributes to clinical deterioration and death. Accordingly, recognition of broad morphologic patterns at necropsy may help prioritize ancillary tests, interpret concurrent diseases, and refine differential diagnoses in comparable antemortem cases, especially in rescued, outdoor-access, or otherwise under-monitored cats. The observation that pneumonia represented the primary cause of death or euthanasia in many cases, but was contributory or incidental in others, further emphasizes the importance of integrating pulmonary pathology with the broader clinicopathological context (1, 14, 16).

As a retrospective necropsy-based investigation, this study was subject to inherent limitations, including incomplete availability of bacterial cultures, restricted viral diagnostic testing, and variable completeness of clinical and archival records. These limitations were particularly relevant because the study population consisted of free-roaming, rescued, and outdoor-access cats, many of which were submitted for necropsy without prior clinical evaluation, with limited antemortem history, or without adequate clinical follow-up. Accordingly, interpretation of the clinicopathological relevance of pneumonia required integration of the available history with gross and histopathological findings, and in some cases the absence of detailed antemortem data may have limited more precise etiologic or outcome-based interpretation. These factors underscore the value of standardized clinical reporting and consistent post-mortem sampling protocols for future studies addressing the etiopathogenesis of feline pneumonia.

## Conclusion

Feline pneumonia in free-roaming cats comprises a heterogeneous group of morphologic patterns, with bronchopneumonia predominating, often in association with important concurrent systemic disease. Certain patterns carry more immediate practical implications: vasculocentric pyogranulomatous pneumonia supports consideration of feline infectious peritonitis, granulomatous pneumonia should prompt targeted histochemical and microbiological investigation, and aspiration or pleuropneumonia identify clinically actionable contexts such as perioperative aspiration or severe pleural disease. More broadly, post-mortem recognition of pulmonary patterns is useful not only for pathologic classification but also for clinicopathological reasoning, interpretation of comorbidities, and refinement of differential diagnoses in under-monitored free-roaming cats.

## Data Availability

The original contributions presented in the study are included in the article/Supplementary material, further inquiries can be directed to the corresponding author.
