# Supplementary material for: Morphological patterns of pneumonia in free-roaming cats: a necropsy and histopathological study
Source: Front Vet Sci. 2026 Jun 25;13:1855974. doi: 10.3389/fvets.2026.1855974 (PMC13347189; doi:10.3389/fvets.2026.1855974)
Supplement: Supplementary file 1 [file Table_1.DOCX]

**Supplementary Table S1. Antemortem clinical context and inferred relevance of pneumonia to outcome in 49 free-roaming cats**

| **Case no.** | **Age category** | **Type of death** | **Antemortem clinical context** | **Inferred relevance of pneumonia to outcome** | **Morphologic pattern of pneumonia** | **Etiologic diagnosis / interpretation (when available)** |
| --- | --- | --- | --- | --- | --- | --- |
| 1 | Adult | Spontaneous death | Presented moribund with dyspnea, cyanosis, and recumbency; died during clinical evaluation. | Primary | Granulomatous pneumonia | *Cryptococcus spp.* |
| 2 | Juvenile | Spontaneous death | Ptyalism, nasal and ocular discharge; multi-cat household exposure. | Primary | Conventional bronchopneumonia | Not determined |
| 3 | Geriatric | Spontaneous death | Submitted dead; no informative clinical history available. | Contributing | Pyogranulomatous pneumonia | FIP-compatible |
| 4 | Adult | Spontaneous death | Submitted dead; no informative clinical history available. | Primary | Pleuropneumonia | Not determined |
| 5 | Adult | Spontaneous death | Rescued from the street in poor general condition, recumbent and soiled with feces and urine; died spontaneously. | Primary | Conventional bronchopneumonia | Not determined |
| 6 | Kitten | Spontaneous death | Submitted dead; no informative clinical history available. | Primary | Interstitial pneumonia | Not determined |
| 7 | Juvenile | Spontaneous death | Cough and respiratory noise for approximately 1 month; hyporexia for 3 days. | Primary | Conventional bronchopneumonia | Not determined |
| 8 | Juvenile | Spontaneous death | Systemic decline with apathy, anorexia, cachexia, and blindness; prior clinical evaluation performed. | Incidental | Conventional bronchopneumonia | Not determined |
| 9 | Adult | Spontaneous death | Weakness, anorexia, ataxia, dry cough, and diarrhea after returning home. | Incidental | Conventional bronchopneumonia | Not determined |
| 10 | Kitten | Spontaneous death | Abdominal pain and gagging before death; diarrhea, hepatic steatosis, and chronic kidney disease. | Incidental | Aspiration pneumonia | Aspiration-associated; concurrent feline panleukopenia |
| 11 | Adult | Spontaneous death | Respiratory distress with recumbency and severe dehydration; postpartum and still lactating. | Primary | Interstitial pneumonia | Not determined |
| 12 | Adult | Spontaneous death | Systemic decline with lethargy and recumbency after 2 days missing; renal cysts and chronic kidney disease. | Contributing | Conventional bronchopneumonia | Not determined |
| 13 | Juvenile | Spontaneous death | Submitted dead; no informative clinical history available. | Contributing | Conventional bronchopneumonia | Not determined |
| 14 | Adult | Spontaneous death | Hepatobiliary disease with inappetence, vomiting, and icterus; hospitalized for 6 days. | Incidental | Interstitial pneumonia | Not determined |
| 15 | Adult | Spontaneous death | Returned home after several days outdoors with sialorrhea, dyspnea, and lateral recumbency. | Primary | Pleuropneumonia | Not determined |
| 16 | Age not recorded | Spontaneous death | Multi-cat household outbreak with fever, ptyalism, dehydration, oral ulcers, leukopenia, and thrombocytopenia; multiple cats died. | Primary | Conventional bronchopneumonia | Bacterial, not further specified |
| 17 | Age not recorded | Spontaneous death | Same multi-cat household outbreak with fever, ptyalism, dehydration, oral ulcers, leukopenia, and thrombocytopenia; multiple cats died. | Primary | Conventional bronchopneumonia | Bacterial, not further specified |
| 18 | Juvenile | Spontaneous death | Respiratory distress with vomiting, reduced appetite, nasal discharge, halitosis, and pleural content. | Primary | Conventional bronchopneumonia | *Klebsiella pneumoniae* |
| 19 | Adult | Spontaneous death | Postoperative decline after recurrent intestinal obstruction; tracheal tube obstruction or aspiration suspected. | Primary | Aspiration pneumonia | Aspiration-associated |
| 20 | Adult | Spontaneous death | Systemic neurologic/septic presentation after overnight outdoor exposure; concurrent meningoencephalitic and septic lesions. | Contributing | Conventional bronchopneumonia | Bacterial, not further specified |
| 21 | Kitten | Spontaneous death | Submitted dead; no informative clinical history available. | Primary | Conventional bronchopneumonia | Not determined |
| 22 | Age not recorded | Spontaneous death | Submitted dead; no informative clinical history available. | Contributing | Pyogranulomatous pneumonia | FIP-compatible |
| 23 | Adult | Spontaneous death | Respiratory distress in severe chronic kidney disease; irregular kidneys and nephrolithiasis on ultrasonography. | Contributing | Conventional bronchopneumonia | Not determined |
| 24 | Adult | Spontaneous death | Submitted dead; no informative clinical history available. | Contributing | Pyogranulomatous pneumonia | FIP-compatible |
| 25 | Adult | Spontaneous death | Submitted dead; no informative clinical history available. | Primary | Aspiration pneumonia | Aspiration-associated |
| 26 | Adult | Spontaneous death | Submitted dead; no informative clinical history available. | Contributing | Pyogranulomatous pneumonia | FIP-compatible |
| 27 | Kitten | Spontaneous death | Submitted dead; no informative clinical history available. | Contributing | Conventional bronchopneumonia | *Escherichia coli* |
| 28 | Adult | Spontaneous death | Submitted dead; no informative clinical history available. | Primary | Conventional bronchopneumonia | Not determined |
| 29 | Adult | Spontaneous death | Submitted dead; no informative clinical history available. | Primary | Conventional bronchopneumonia | Not determined |
| 30 | Adult | Spontaneous death | Respiratory distress after parturition with anorexia; thoracic radiography showed increased pulmonary opacity and a prominent bronchial pattern. | Primary | Bronchointerstitial pneumonia | Not determined |
| 31 | Adult | Spontaneous death | Submitted dead; no informative clinical history available. | Primary | Interstitial pneumonia | Not determined |
| 32 | Geriatric | Spontaneous death | Respiratory distress; oxygen therapy performed before death. | Primary | Conventional bronchopneumonia | *Staphylococcus spp.* |
| 33 | Juvenile | Spontaneous death | Submitted dead; no informative clinical history available. | Primary | Bronchointerstitial pneumonia | Not determined |
| 34 | Adult | Spontaneous death | Submitted dead; no informative clinical history available. | Contributing | Conventional bronchopneumonia | Not determined |
| 35 | Kitten | Spontaneous death | Submitted dead; no informative clinical history available. | Contributing | Conventional bronchopneumonia | *Escherichia coli* |
| 36 | Adult | Spontaneous death | Submitted dead; no informative clinical history available. | Primary | Conventional bronchopneumonia | Not determined |
| 37 | Adult | Spontaneous death | Submitted dead; no informative clinical history available. | Primary | Conventional bronchopneumonia | Not determined |
| 38 | Adult | Euthanasia | Respiratory and pulmonary disease suspected on radiography, with pneumoperitoneum and severe systemic abnormalities on ultrasonography and CBC; euthanized. | Primary | Conventional bronchopneumonia | Bacterial, not further specified |
| 39 | Adult | Spontaneous death | Respiratory distress with fever, apathy, vomiting, and pleural disease; thoracocentesis yielded turbid pleural fluid. | Primary | Pleuropneumonia | *Staphylococcus aureus* |
| 40 | Adult | Euthanasia | Severe respiratory distress in a cachectic, dehydrated rescued cat; manual ventilation attempted before euthanasia. | Primary | Conventional bronchopneumonia | Mixed bacterial infection (*Klebsiella pneumoniae*, *Pseudomonas spp.*, and *Staphylococcus aureus*) |
| 41 | Adult | Spontaneous death | Progressive systemic disease with cachexia, chronic limb edema, pain, hypertrophic osteopathy, and radiographic nodules suggestive of granulomas. | Primary | Granulomatous pneumonia | Mycobacterial infection |
| 42 | Adult | Spontaneous death | Progressive upper respiratory neoplasia with nasal mass involving the nasal planum and lip. | Contributing | Conventional bronchopneumonia | Not determined |
| 43 | Kitten | Spontaneous death | Respiratory distress with neurologic signs and an alveolar radiographic pattern in a rescued kitten. | Contributing | Pyogranulomatous pneumonia | FIP-compatible |
| 44 | Adult | Spontaneous death | Systemic and perioperative decline with anorexia, vomiting, weakness, dysuria, dehydration, and pale mucous membranes. | Contributing | Interstitial pneumonia (mixed bronchopneumonic and interstitial pattern) | *Aelurostrongylus abstrusus* with secondary *Klebsiella aerogenes* |
| 45 | Kitten | Spontaneous death | Traumatic thoracoabdominal disease with incidental diaphragmatic hernia, pulmonary contusion, and pneumopathy. | Contributing | Granulomatous pneumonia | *Nocardia spp.* |
| 46 | Adult | Spontaneous death | Respiratory distress with collapse and nasal discharge; oxygen therapy, fluid therapy, and ampicillin administered. | Primary | Conventional bronchopneumonia | Enterobacter spp. |
| 47 | Adult | Spontaneous death | Respiratory distress with anorexia and mild icterus; FeLV-positive. | Primary | Interstitial pneumonia | Not determined |
| 48 | Kitten | Spontaneous death | Respiratory distress documented on the day before presentation. | Primary | Conventional bronchopneumonia | *Escherichia coli* |
| 49 | Juvenile | Spontaneous death | Severe respiratory distress with oxygen dependence in a cat with intestinal intussusception. | Primary | Embolic pneumonia | Septic embolic bacterial pneumonia associated with intestinal intussusception |
